# Electrocatalysis in deep eutectic solvents: from fundamental properties to applications

**DOI:** 10.1039/d4sc02318h

**Published:** 2024-06-05

**Authors:** Hengan Wang, Xinchen Kang, Buxing Han

**Affiliations:** a Beijing National Laboratory for Molecular Sciences, CAS Laboratory of Colloid and Interface and Thermodynamics, CAS Research/Education Centre for Excellence in Molecular Sciences, Centre for Carbon Neutral Chemistry, Institute of Chemistry, Chinese Academy of Sciences Beijing 100190 China hanbx@iccas.ac.cn; b School of Chemistry, University of Chinese Academy of Sciences Beijing 100049 China; c Shanghai Key Laboratory of Green Chemistry and Chemical Processes, School of Chemistry and Molecular Engineering, East China Normal University Shanghai 200062 China

## Abstract

Electrocatalysis stands out as a promising avenue for synthesizing high-value products with minimal environmental footprint, aligning with the imperative for sustainable energy solutions. Deep eutectic solvents (DESs), renowned for their eco-friendly, safe, and cost-effective nature, present myriad advantages, including extensive opportunities for material innovation and utilization as reaction media in electrocatalysis. This review initiates with an exposition on the distinctive features of DESs, progressing to explore their applications as solvents in electrocatalyst synthesis and electrocatalysis. Additionally, it offers an insightful analysis of the challenges and prospects inherent in electrocatalysis within DESs. By delving into these aspects comprehensively, this review aims to furnish a nuanced understanding of DESs, thus broadening their horizons in the realm of electrocatalysis and facilitating their expanded application.

## Abstract 2

This review provides a comprehensive summary of the characteristics of DESs and their utility as solvents for electrocatalyst synthesis and as electrolytes in typical electrocatalytic reactions.

## Graphical abstract

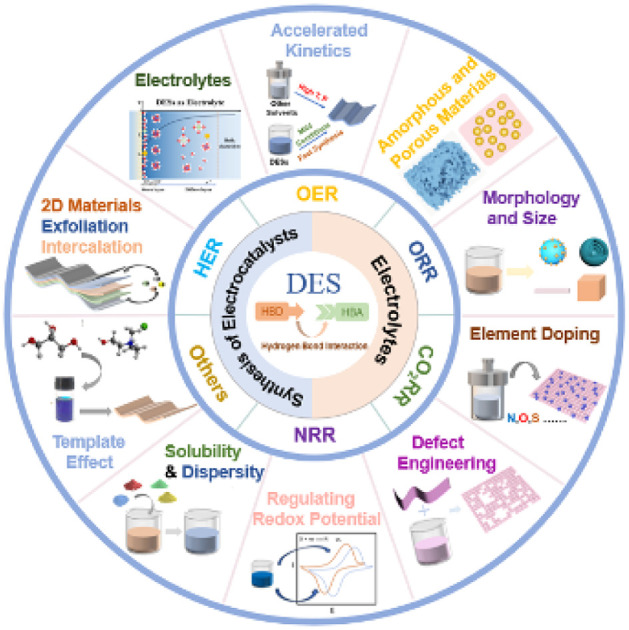

## 1. Introduction

The excessive utilization of fossil fuels has brought about a growing energy crisis and environmental dilemma for mankind.^1–5^ The exploration and development of renewable energy sources aimed at mitigating global energy and environmental challenges has come to the forefront. Electrocatalysis stands out as a strong contender among various strategies of clean energy transformations, showing excellent capacity to transform molecules into higher-value products under mild conditions.^6–10^ For instance, the oxygen reduction reaction (ORR) and oxygen evolution reaction (OER) represent pivotal half-reactions in rechargeable metal–air batteries, and the hydrogen evolution reaction (HER) plays a critical role in the comprehensive electrocatalytic water splitting.^11–16^ Additionally, the electrocatalytic CO_2_ reduction reaction (CO_2_RR) and nitrogen reduction reaction (NRR) have attracted extensive attention as CO_2_ and N_2_ are ample resources.^17–23^

**Fig. 1:**
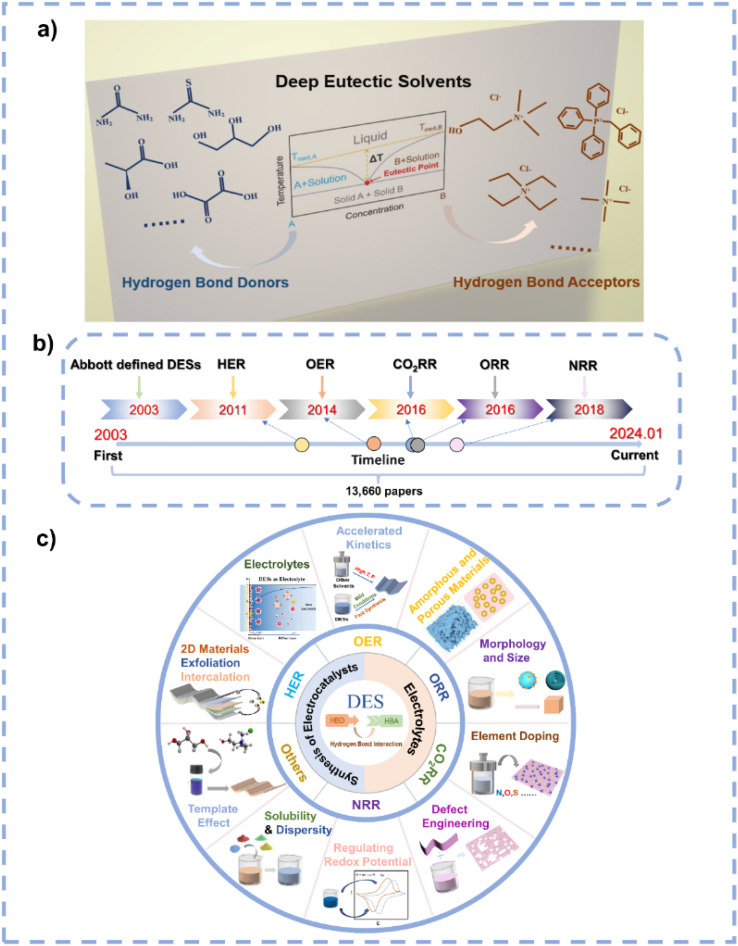
(a) Prototypical binary phase diagram for DESs and some representative examples of HBDs and HBAs. (b) The development of DESs in electrocatalysis. (c) Applications of DESs in electrocatalysis.

**Fig. 2:**
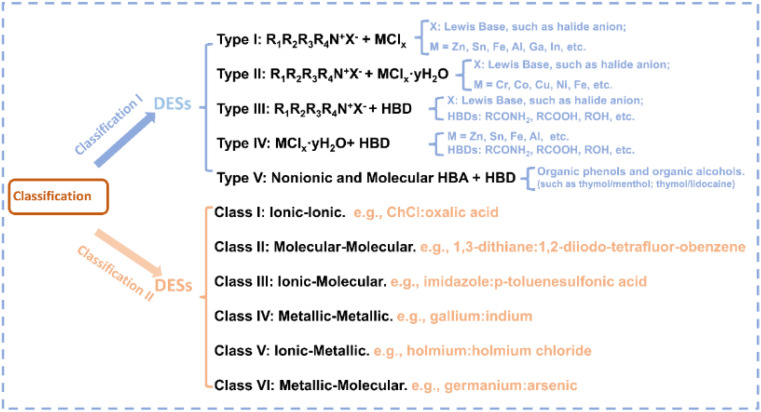
The classification of DESs in two different ways.

DESs represent environmentally friendly liquid media formed by the self-association of two or three components through hydrogen bond interactions between hydrogen bond donors (HBDs) and acceptors (HBAs) (Fig. 1a).^24^ The term “eutectic” was first proposed in 1884 to indicate “a lower temperature of liquefaction than that given by any other proportion”;^25^ the term “deep” refers to a difference between the actual eutectic temperature and the ideal eutectic temperature in the phase diagram when the ideal eutectic temperature is higher than the actual eutectic temperature.^26,27^ In 2003, Abbott *et al.* discovered an abnormal decrease in the melting point at the eutectic composition of urea and choline chloride (ChCl).^28^ The early DESs are characterized by ionic HBAs and HBDs, after which they are further expanded to non-ionic molecular HBAs and HBDs,^29^ and they are categorized into five distinct types in the early research (Fig. 2).^30^ Recently, Chen *et al.* proposed a new classification of DESs including ionic–ionic, molecular–molecular, ionic–molecular, metallic–metallic, ionic–metallic, and molecular–metallic types.^26^ In the past decades, DES research has received broad attention, with a large increase in the number of relevant research articles per year (Fig. 1b). As DESs show unique characteristics including simple preparation, cost-effectiveness, low vapor pressure, nonflammability, excellent solubility and dispersibility, high thermal and chemical stability, high ionic conductivity, wide electrochemical stable window (ESW), designability, compositional tunability, *etc.*,^30–40^ they have been widely used in the fields of extraction and separation,^41,42^ gas capture and separation,^43–45^ batteries,^31,40,46,47^ energy storage,^48–50^ materials preparation,^33,51–53^ and catalysis.^54,55^

Solvents play significant roles in material synthesis and chemical reactions as they can modulate the nucleation and growth of materials and the pathways of reactions.^56,57^ Due to their special characteristics, DESs are potential solvents to control the synthesis of materials and chemicals. Particularly, DESs are suitable candidates in electrocatalysis because of their high conductivity, wide ESW, and high stability, and they can be employed as solvents for the synthesis of electrocatalysts as well as being used as electrolytes. However, the current research on DESs related to electrocatalysis is very limited (Fig. 1b). Therefore, a prompt and comprehensive review on the application of DESs in electrocatalysis is highly desirable. This review first summarizes the features of DESs. Subsequently, the distinct advantages of DESs as solvents/electrolytes in electrocatalysis are highlighted, and the unique functions of DESs in electrocatalyst synthesis and electrocatalysis are detailed (Fig. 1c). Finally, this review examines the challenges and opportunities of DESs in electrocatalysis. Such an overview aims to contribute to the development of this emerging field and to advance the understanding of the role of DESs in electrocatalysis.

## 2. Features of DESs applicable to electrocatalysis

### 2.1 Interactions

**Fig. 3:**
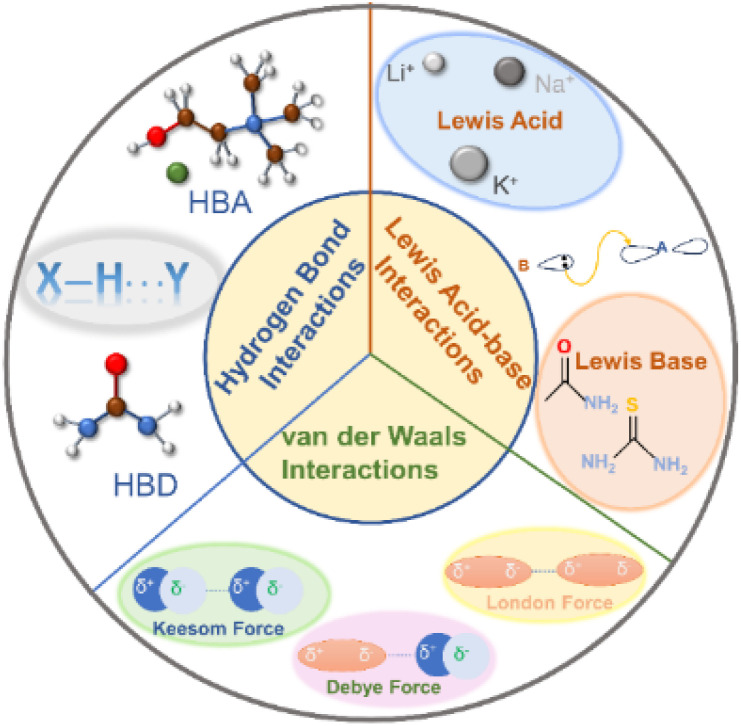
Interactions in DESs.

DESs are composed of at least two components, each of which has a melting point higher than the final mixture. Their formation is dependent on intermolecular interactions among diverse components, surpassing the inherent interactions within each component.^39^ Typically, three primary interactions drive the formation of DESs, as illustrated in Fig. 3.

The foremost interaction is hydrogen bonding, which mainly determines the formation of DESs. Such DESs encompass at least one HBA [*e.g.* ChCl, metal halides, and quaternary ammonium salt analogous] and an HBD (*e.g.* amide, alcohol, or carboxylic acid molecules).^39^ The robust hydrogen bond between the HBA and the HBD reduces their original intermolecular interactions, resulting in a reduced melting point.^24,39^ Furthermore, the hydrogen bond determines the eutectic point of the HBD to the HBA.^39^ For instance, the eutectic points of ChCl/urea and ChCl/thiourea systems are 1/2 (n/n) and 1/3 (n/n), respectively.^58^ Thus, varying the molar ratio of HBDs and HBAs selection allows for finer control over the physicochemical properties of DESs.

Lewis acid–base interaction is another important interaction in DES systems. A Lewis acid is defined as a substance with the capability to accept an electron pair, which is donated by a Lewis base. Under the Lewis acid–base theory, cations as well as compounds such as BF_3_ that could accept electron pairs can act as Lewis acids while anions as well as compounds like NH_3_ and PH_3_ containing lone electron pairs are considered as Lewis bases. Indeed, Lewis acid–base interactions in DESs are not considered sufficient for the formation of covalent bonds.^39^ They are preferable to only change the coordination environment to obtain a low-melting point eutectic solvent.^39^ Li^+^, Na^+^, and K^+^ are typical Lewis acids and can form DESs with a variety of Lewis bases such as amides, carbonates, and ethers.^39,59^

van der Waals interactions, comprising Keesom forces (dipole–dipole effect), Debye forces (dipole–induced dipole effect), and London dispersion forces, widely exist in DESs.^39,60^ Keesom force emerges from the interaction of permanent dipoles; Debye force is the attractive energy between a polar molecule and a nonpolar molecule; London dispersion force refers to the weak attractive force between the instantaneous dipole moments of molecules when they are close to each other. van der Waals interaction is the weakest interaction among chemical forces, possessing a strength of about 4 kJ mol^−1^.^61^ van der Waals interactions are weak, yet they can occur and show up between any two molecules, which makes them essential to the whole system. Generally, van der Waals interactions predominate in eutectic systems, which manifests that the original intermolecular interactions of the single component are replaced by the newly produced van der Waals forces after adding a new component.^39^ Notably, van der Waals forces play a key role in fine-tuning the physicochemical properties of DESs, working together with other interactions.

The synergy of hydrogen bonds, Lewis acid–base interaction, and van der Waals influences the formation and properties of DESs. The intermolecular interactions can be further modified by changing the functional group of molecules and the composition of the mixture to determine the properties and phase behavior of DESs. The presence of versatile interactions in DESs significantly increases the solubility for small molecules, which is favorable for the adsorption and conversion of small molecules in electrocatalytic reactions.^62–65^ Besides, interactions in DESs could construct supramolecular networks to control the growth direction of materials and could also play significant roles in activating reactants and stabilizing intermediates,^30,40,66–68^ making DESs promising candidates as electrolytes for electrocatalysis.

### 2.2 Physicochemical properties

The interactions in DESs determine the physicochemical properties of DESs. Their unique physicochemical features enable precise control over electrocatalyst production and electrocatalysis. For example, the vapor pressure of DESs is typically low,^69^ which not only reduces the solvent loss during the reaction but also allows for a liquid state at a wide temperature range, enhancing the applicability across diverse environmental conditions. Despite the conductivity of independent DESs at room temperature being low due to the high viscosity, changing the temperature or introducing water molecules into DESs could produce high-conductivity DES systems as a large number of highly mobile ions exist in DESs.^70–72^ DESs have a wide ESW, which results in the high electrochemical stability of DESs in electrocatalytic reactions.^73,74^ Variations in the molar ratio of eutectic components can alter the overall ESW, allowing for tailoring electrochemical performance.^74^ The wide ESW provides a broad prospect for DESs as electrolytes to manipulate the electric double layer (EDL). The inherent ionic properties and relatively high polarity of DESs result in their good solubility that could dissolve various inorganic and organic compounds,^24^ and the Lewis acid/base sites or hydrogen bonds in DESs can bind with solutes to promote their dissolution. The excellent dissolving ability makes DESs suitable solvents for materials synthesis and chemical reactions.^75–79^ The viscosity of DESs significantly exceeds that of typical molecular solvents, which leads to low current densities when DESs are used as electrolytes.^80^ However, the viscosity could be reduced by adding extra molecules, using small ions, and increasing temperature in terms of Hole theory.^24,58^ The difference in the freezing point at the eutectic composition of a binary mixture of A + B compared to that of a theoretical ideal mixture (Δ*T*) is an important parameter concerning the phase behavior of DESs, and the stronger interaction between HBDs and HBAs results in a higher Δ*T* value,^58^ which extends the liquid range of DESs as electrolytes. DESs can mediate the synthesis of materials and the microenvironment of EDL through its role in pH regulation.^81,82^ The pH of DESs is closely related to temperature and the ratio of HBDs to HBAs and influences the EDL microenvironment.^81,82^ Thus, electrocatalyst synthesis could be modulated by tuning the pH of DESs.^24^ High entropy is one of the unique thermodynamic properties of DESs. For the formation of DESs [A (solid) + B (solid) → A–B (liquid)], the entropy change of the above processes should be positive because both solid–liquid transformation and mixing processes increase the entropy of the system.^26^ Inspired by the concept of high-entropy alloys,^83,84^ DESs could be high-entropy DESs.^26^ Moreover, the presence of two or more compositions of HBAs and HBDs provides the possibility for the formation of high entropy solvents, which are favorable for modulating the solvation structure and enhancing the ionic conductivity.^85–87^

## 3. DESs as solvents

### 3.1 Advantages of DESs as solvents

**Fig. 4:**
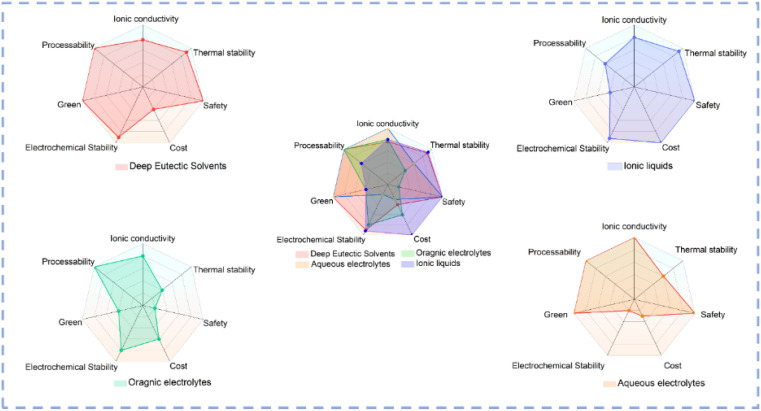
Radar plots of various solvents.^31,93–95^

Electrode materials and electrolytes are two main components in a typical electrocatalytic system. DESs could not only be directly used as electrolytes but also be used as solvents for the controlled synthesis of electrocatalysts. Fig. 4 illustrates a comparative analysis of the properties of various solvents used as electrolytes.^88^ In conventional aqueous electrolyte systems, the narrow ESW results in poor electrochemical stability, and serious HER always occurs which renders a decreased selectivity toward the target product.^89–91^ As organic solvents have very low ionic conductivity, supporting electrolytes such as ionic liquids (ILs) are usually introduced into the solvent to prepare organic electrolytes.^88,92^ Although they exhibit high conductivity, the environmentally unfriendly organic solvent and high cost of the supporting electrolyte limit the large-scale applications of organic electrolytes in electrocatalysis. Compared with aqueous and organic systems, DESs exhibit unique advantages such as low cost, high stability, environmental friendliness, *etc.* The distinctive hydrogen bond structure of DESs plays a pivotal role in regulating the EDL structure to modulate the ionic solvation environment and stabilize intermediates, thus affecting the selectivity and kinetics of electrocatalytic reactions. Due to their excellent dissolving ability, low vapor pressure, and high stability, DESs could be used as solvents for material synthesis at high temperature. The hydrogen bond network and electrostatic interaction in DES systems create a special microenvironment distinct from conventional solvents, and the unique microenvironment can not only modulate the reaction kinetics but also change the thermodynamic behavior of the solution. DESs exhibit unique advantages as solvents for electrocatalyst synthesis and as electrolytes (Table 1), which are summarized in detail in the subsequent section.

**Table 1:** The advantages of DESs in electrocatalysis

Solvents for electrocatalyst synthesis	Electrolyte for electrocatalytic reactions
Excellent dissolving and dispersing ability	High reactant solubility
Accelerated kinetics	Rapid electron transfer rate
Morphology-structure control	Controllable ion solvation environment
Elemental doping	Tunable EDL structure
Defect engineering	Reduced overpotential
Regulating redox potential	Enhanced stabilization of key intermediates
Intercalating and exfoliating	Strong interactions with reactants or products

### 3.2 DESs as solvents for electrocatalyst synthesis

#### 3.2.1 Dissolving and dispersing

**Fig. 5:**
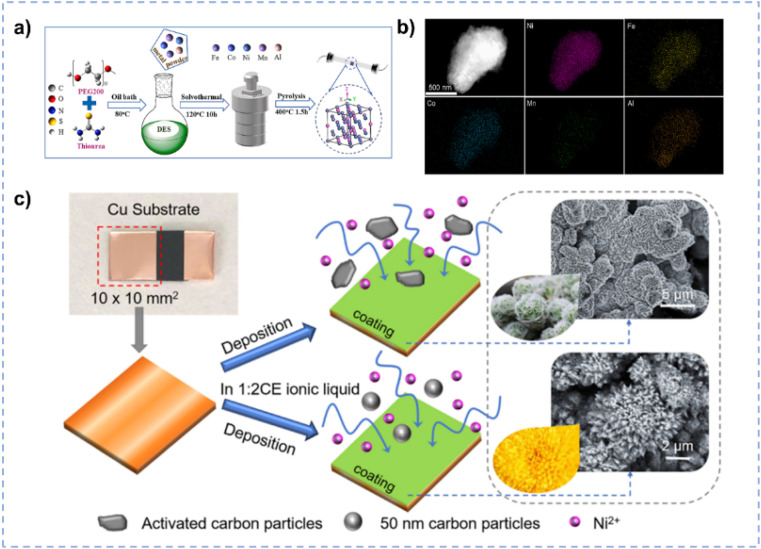
(a) Preparation of high-entropy materials in DES. (b) EDS elemental maps of the NiFeCoMnAl high-entropy material. Reprinted with permission from ref. 102 Copyright 2024, Elsevier. (c) Electrodeposition of Ni matrix composite coatings on the Cu substrate in ChCl/EG and aqueous electrolytes, respectively. Reprinted with permission from ref. 111 Copyright 2019, Elsevier.

DESs have a strong ability to dissolve precursors and disperse materials.^96–101^ DESs can be used as excellent solvents for hydrothermal synthesis of high-entropy alloy NiFeCoMnAl due to the good solubility of all precursors including MnCl_2_, NiCl_2_, CoCl_2_, FeCl_3_, and AlCl_3_ (Fig. 5a).^102^ DESs can disperse individual metals uniformly which promotes the formation of high-entropy materials (Fig. 5b). Furthermore, the microscopic morphology of high-entropy alloys can be precisely modulated by tuning distinct HBAs and HBDs such as urea, thiourea, citric acid, polyethylene glycol (PEG), and ethylene glycol (EG). Among them, NiFeCoMnAl synthesized in PEG/thiourea shows excellent OER performance with an overpotential of only 220 mV and 317 mV at 10 mA cm^−2^ and 100 mA cm^−2^, respectively. DESs also exhibit remarkable capabilities in dissolving and capturing gas molecules.^103–105^ The solubility of gas molecules in the electrolyte significantly influences the adsorption and mass transport, consequently impacting the performance of electrocatalysis.^106^ For example, ChCl/EG with a molar ratio of 1 : 2 exhibits excellent dissolving ability for CO_2_ (1.3 mol CO_2_ per mol ChCl/EG; 329 K, 76.5 kPa), resulting in the boosted CO_2_ electro-reduction performance.^107^ Protsenko *et al.* synthesized Ni/TiO_2_ by electrodeposition in ChCl/EG.^108^ Through intensifying the stirring of ChCl/EG, the transfer of colloidal particles to the electrode surface is accelerated, and hence more dispersed particles can be electrodeposited on the electrode surface. The as-prepared Ni/TiO_2_ exhibits enhanced electrocatalytic properties for the HER, compared with that prepared in conventional aqueous electrolytes. The homogeneous dispersion of second-phase particles in the electrolyte is crucial to obtain the uniform coating during the electro-deposition.^109,110^ Wang *et al.* prepared a three-dimensional flower-like structure of nickel matrix composite electrodes *via* facile electrodeposition in ChCl/EG for the HER (Fig. 5c).^111^ The high viscosity of ChCl/EG is conductive to diminish the settling velocity of particles and the high ionic strength reduces the interactions among particles, preventing extensive particle agglomeration within the electrolyte.^110^

#### 3.2.2 Accelerated kinetics

The kinetics for the material formation could be accelerated in DESs.^30,112^ This mainly stems from the homogeneous medium provided by their hydrogen-bonding framework and the template effect, as well as energy reduction effects due to their supramolecular properties. Moreover, DESs possess excellent solvation properties and are able to better dissolve reactants, which helps to increase the effective concentration of reactants, improve the reaction rate, and promote collisions and reactions between reactants. Traditionally, cobalt–vanadium oxides are prepared by solid-state reaction, hydrothermal reaction, or co-precipitation methods with a long reaction time and high temperature.^113,114^ However, octahedral CoV_2_O_6_ nanomaterials could be well synthesized under mild conditions in the ChCl/malonic acid system.^30,112^ Söldner *et al.* synthesized spinel-type ferrite nanoparticles MFe_2_O_4_ (M = Mg, Zn, Co, Ni) in ChCl/maleic acid.^115^ The synthesis of MFe_2_O_4_ in the DES could proceed at much lower temperatures than other methods, and the resultant materials show comparable properties with those synthesized by other strategies.^115^ In addition to facilitating the synthesis of inorganic materials, kinetics for the synthesis of organic materials such as covalent organic frameworks (COFs) can also be accelerated.^116^ Qiu *et al.* prepared 2D and 3D-COFs in the ChCl/glycerol system.^117^ The 2D-COF TpPA (1,3,5-triformylphloroglucinol (Tp); *p*-phenylenediamine (PA)) could be generated in a gram-scale at room temperature after 2 h in DES. In contrast, the synthesis of TpPA requires a temperature of 120 °C for 3 days by a solvothermal reaction.^118^

#### 3.2.3 Morphology control

**Fig. 6:**
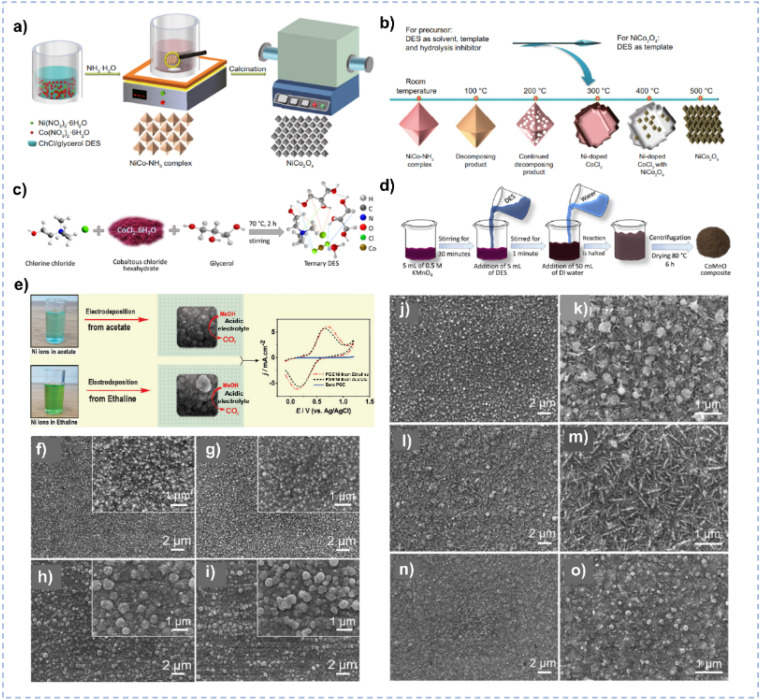
(a) Synthesis process of the NiCo–NH_3_ complex and NiCo_2_O_4_ in ChCl/glycerol. (b) The effect of DES and calcination temperature on the composition and morphology of NiCo_2_O_4_. Reprinted with permission from ref. 120 Copyright 2022, Elsevier. (c) The formation of the ternary-component DES. (d) Synthesis of CoMnO in ChCl/CoCl_2_/glycerol ternary DES. Reprinted with permission from ref. 121 Copyright 2023, American Chemical Society. (e) Electrodeposition of Ni nanoparticles in different solvents. Reprinted with permission from ref. 125 Copyright 2021, Elsevier. (f–i) SEM images of the Ni films synthesized in ChCl/urea at −0.6 V (f), −0.7 V (g), −0.8 V (h), and −0.9 V (i) at 328 K for 1 h. The insets are amplified SEM images. (j–o) SEM images of the Ni films synthesized in ChCl/EG at −0.5 V (j, and k), −0.6 V (l, and m) and −0.7 V (n, and o) at 343 K for 1 h. Reprinted with permission from ref. 126 Copyright 2018, Elsevier.

The complicated interactions render various phase or aggregation behavior in DES systems,^33^ and the domains generated in DESs could be employed as soft templates for modulating the micro/nano-structure of materials. In 2004, Cooper *et al.* pioneered an innovative approach for fabricating porous solids, utilizing DESs as solvent and structure-directing agents.^119^ Recently, Zhang *et al.* reported a DES-mediated strategy to synthesize an octahedral NiCo–NH_3_ complex, which could be directly transformed into NiCo_2_O_4_ nanooctahedrons after thermal decomposition (Fig. 6a).^120^ The NiCo–NH_3_ precursor in octahedral shape is achieved with the DES-mediated crystallization in ChCl/glycerol.^120^ The utilization of DES, ChCl/glycerol, not only customizes the morphology of the prepared precursor *via* a template effect but also effectively restrains its hydrolysis, guaranteeing the successful synthesis of the octahedral NiCo–NH_3_ complex with a high yield (Fig. 6b). In addition to acting as single template agents, DESs can also play multifunctional roles. For instance, a ternary-component DES, ChCl/CoCl_2_·6H_2_O/glycerol, is employed as the reaction medium, reducing agent, template, and metal source in the room-temperature synthesis of the CoMnO compound (Fig. 6c).^121^ The hydrogen-bonding interaction between the DES and manganese precursor facilitates the expeditious formation of CoMnO nanostructures. Moreover, this synthesis process and hydrogen-bonding interaction could be regulated by the introduction of water, and water molecules can weaken the bonding between the formed nanostructures and ultimately affect the morphology of CoMnO (Fig. 6d).

In addition, nanomaterials with a smaller particle size, have a larger surface area and tend to supply more active sites during electrocatalysis.^122–124^ DESs could be used as solvents for the controlled electrodeposition to synthesize electrocatalysts of small particle size. Ni nanoparticles synthesized by electrodeposition in ChCl/EG exhibit a smaller particle size and more uniform morphology compared with those synthesized in an acetate buffer solution owing to the coordination between Ni^2+^ and ChCl/EG (Fig. 6e),^125^ leading to the higher catalytic performance for electrooxidation of methanol. The composition of DESs could also influence the morphology of as-synthesized materials. ChCl/urea and ChCl/EG DESs yield distinct Ni films, attributable to their disparate growth processes and assembly behaviors.^126^ Notably, ChCl/EG exhibits lower viscosity and higher conductivity in comparison to ChCl/urea. The diffusion coefficient of Ni^2+^ species within ChCl/urea manifests a considerably slower rate than that observed in ChCl/EG, and Ni^2+^ exhibits different coordination behavior in the two DESs. Thereby, the Ni electrodeposit obtained in ChCl/EG exhibits a smaller particle size than that produced in ChCl/urea (Fig. 6f–o).^126^

#### 3.2.4 Structure control

The structure of electrocatalysts normally determines their performance in electrocatalysis. For example, amorphous electrocatalysts have reduced coordination number distribution, abundant surface dangling bonds, and broadened energy bands, contributing to optimizing catalytically active sites and reducing overpotential during electrocatalysis.^127^ Porous materials possess a large specific surface area and a large number of active sites that facilitate the adsorption, activation, and transformation of reactants.^128–130^

DESs have a large supramolecular hydrogen-bond network structure formed by HBDs and HBAs, which inhibits the crystallization of materials to generate an amorphous structure.^131^ Jang *et al.* prepared amorphous CaCO_3_ in an alkanolamine-based DES–H_2_O system.^131^ The experimental results show that the strength of the hydrogen bond is the main factor affecting the solvent properties in the DES–H_2_O system. Moreover, the DES–H_2_O system with inter- and intramolecular hydrogen bonds inhibited the crystallization of CaCO_3_, resulting in the formation of amorphous nanostructures. Zhao *et al.* synthesized Ni_2_P supported on Ni_3_(PO_4_)_2_–Ni_2_P_2_O_7_ (Ni_2_P/NiPO) by the reaction of Ni^2+^ and H_2_PO_2_^−^ in ChCl/urea. The Ni_2_P/NiPO material exhibits a surface area of approximately 210 m^2^ g^−1^ with a mesoporous and amorphous structure.^132^ Yang *et al.* prepared self-supported 3D quasi-amorphous Co–O and Co–Se hybrid films on the Cu substrate (Co–O@Co–Se/Cu) *via* a facile one-step electrochemical deposition strategy in ChCl/urea.^133^ The resulting 3D nanostructure with a high surface area and porous architecture increases the number of surface active sites and promotes the intrinsic catalytic activity for the OER. Yang *et al.* employed the ChCl/EG solvent in combination with an electrochemical activation strategy to fabricate monolithic 3D nanoporous Ag/Pd core/shell hybrids with an ultrathin (<1 nm) amorphous Pt-rich skin (Pt–Pd@NPA).^134^ The Pt–Pd@NPA shows exceptional catalytic performance towards the HER across a wide range of pH, attributable to the highly integrated 3D nanoporous architecture and designable electronic structure by the deliberate incorporation of Pt into the interfaces of Ag–Pd hybrids.

**Fig. 7:**
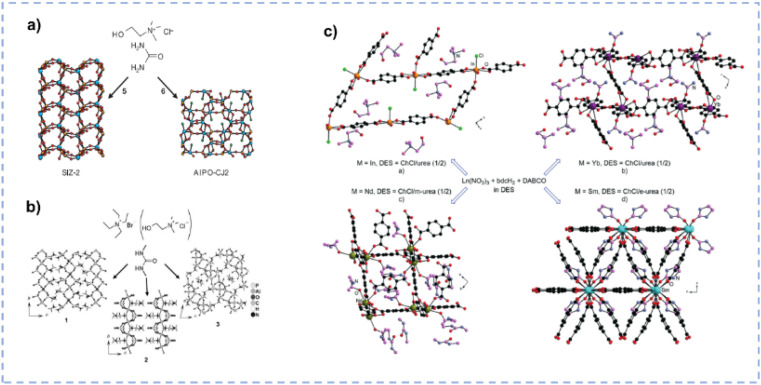
(a) Synthesis of SIZ-2 and AlPO-CJ2 in ChCl/urea. Reprinted with permission from ref. 119 Copyright 2004, Springer Nature Limited. (b) Synthesis of aluminophosphate materials in dimethyl urea based eutectic mixtures with tetraethylammonium bromide or ChCl systems. Reprinted with permission from ref. 135 Copyright 2006, John Wiley and Sons. (c) Structure of MOFs synthesized in different DESs. Reproduced from ref. 116 with permission from the Royal Society of Chemistry.

DESs may partially decompose when the temperature exceeds the pyrolysis temperature of a single component, and the as-generated new species in DESs boost the formation of the porous structure.^33^ The synthesis of a novel zeotype framework [SIZ-2, Al_2_(PO_4_)_3_–3NH_4_] using the ChCl/urea DES as a template and solvent was reported by Cooper *et al.* (Fig. 7a).^119^ The partial decomposition of urea produces ammonia to template the porous structure and balance the charge on the framework, facilitating the formation of the interrupted structure of SIZ-2. Parnham *et al.* synthesized various zeolites in different urea derivative-containing DESs (Fig. 7b).^135^ As anticipated, the thermal degradation of diverse urea derivatives within DESs at elevated temperatures results in the formation of different organic species, which not only act as templates but also facilitate the delivery of reactants. During the synthesis of metal–organic frameworks (MOFs), DES components or decomposition products formed under ionothermal conditions may be involved in the MOF architecture, either being coordinated as a ligand to the metal centres or located in the pores of the MOF host.^116^ Zhang *et al.* synthesized a series of MOFs with the 1,4-benzenedicarboxylate (bdc) ligand in different ChCl/urea derivative (urea/*N*,*N*′-dimethylurea(m-urea)/2-imidazolidinone(e-urea)) mixtures, and DESs play vital roles in controlling the structure of these crystalline materials (Fig. 7c).^136^ For the MOF (Ch)[InCl(bdc)_3/2_(H_2_O)_2_] synthesized in ChCl/urea, the chloride anion is coordinated to the In(iii) cation, and the cholinium cation resides within the pores of the 2D network. In (Ch)[Yb(bdc)_2_(urea)], the cholinium cation is situated within the pores of the 3D architecture, with urea coordinated to the central metal. [Nd(bdc)_2_(Ch)](m-urea) is prepared in ChCl/m-urea, and the cholinium cation coordinates with the metal centre, while m-urea remains unbound within the pores of the 3D structure. As for [Sm(bdc)_3/2_(e-urea)] synthesized in ChCl/e-urea, the e-urea serves as a bridge that connects with Sm(iii) cations, and it can be eliminated by heating the MOF to 300 °C without any structural change. The composition of DESs can mediate the synthesis of MOF materials and ultimately affect the structure, and the rational design of solvents can effectively determine the structure–performance relationship and ultimately affect the properties of the materials.

#### 3.2.5 Defect engineering

**Fig. 8:**
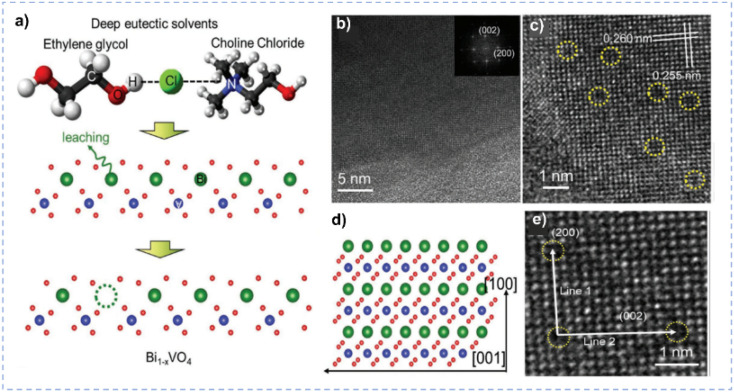
(a) Schematic diagram for the Bi leaching from BiVO_4_ lattices in DES. (b and c) TEM and HRTEM images of Bi_1−*x*_VO_4_. (d) Schematic diagram of the (010) in-plane lattice of BiVO_4_ oriented with respect to the lattice in the TEM image. (e) Enlarged HRTEM image of Bi_1−*x*_VO_4_. Reprinted with permission from ref. 140 Copyright 2021, John Wiley and Sons.

The introduction of defects in electrocatalysts not only tunes the electronic structure of the catalyst to enhance the intrinsic activity but also provides a large number of unsaturated sites to improve electrocatalytic performance.^137–139^ The molecular polarity, hydrogen-bonding interaction, and high elemental extraction ability make DESs suitable solvents for building surface defects of materials. Lu *et al.* leached Bi atoms from the BiVO_4_ lattice to fabricate surface defects by directly immersing BiVO_4_ into ChCl/EG and heating it at different temperatures (Fig. 8a–e).^140^ Thanks to the good metal solubility of DESs, when BiVO_4_ is immersed in ChCl/EG, it leads to partial metal dissolution in DESs and thus builds cationic defects. Theoretical calculations indicate that the Bi–O bond is destroyed by the DES to harvest monoclinic BiVO_4_. The charge transport capacity and carrier separation efficiency of Bi_1−*x*_VO_4_ are effectively improved by this approach.

#### 3.2.6 Element doping

Element doping could be easily realized in DES systems as DESs could not only dissolve various precursors but also serve as precursors directly to prepare doped materials. Sun *et al.* reported the direct electrodeposition of NiP_*x*_ films on Cu foil in a ChCl/EG-based DES.^141^ Within the ChCl/EG-based DES, P is effectively incorporated into Ni superstructures, thereby enabling the NiP_*x*_ hybrid films to obtain a greater number of active sites and enhanced electronic conductivity, which accelerates electron-transfer kinetics for the HER. The ChCl/oxalic acid (ChCl/OA) DES favors doping because it has an excellent dissolving capacity for metal ions and OA can coordinate with various metal ions.^115,142^ Ying *et al.* synthesized Mn-doped Bi_2_O_3_ for the NRR using ChCl/OA as the solvent. Mn-doped Bi_2_O_3_ nanosheets with mesoporous structure have abundant active sites on the surface, which is favorable for mass transfer.^143^ In addition to promoting element doping as solvents, DESs themselves could be used as the source of heteroatoms to prepare doped electrocatalysts. Mou *et al.* synthesized a defect-rich, ultrathin, and tri-doped N, S, O–Co_9_S_8_ in CoCl_2_·6H_2_O/thiourea, and the decomposition of thiourea in the DES during pyrolysis provides the S source and generates a substantial quantity of gases (such as NH_3_, CO_2_, *etc.*) that facilitate the doping of N and O.^144^ Jaihindh *et al.* presented a straightforward strategy to introduce Cl into the CuO lattice using ChCl/urea as the solvent.^145^ The incorporation of Cl into CuO is achieved at ambient temperature through the modulation of molar ratios between ChCl and urea (0.5 : 1, 1 : 1, 1.5 : 1, and 2 : 1). ChCl serves as the source for Cl^−^ anions, and the doping could be modulated by changing the molar ratio of ChCl and urea.^145^

#### 3.2.7 Regulating redox potential of metals

DESs are able to regulate the redox potential of metals, leading to different metal activity sequences compared to those observed in aqueous solutions.^146^ Nickel (*E*^0^ = −0.257 V *vs.* SHE) cannot normally be deposited onto copper (*E*^0^ = 0.34 V *vs.* SHE) without chemical reducing agents in an aqueous solution.^147^ However, experimental findings indicate that the redox potential of Ni^2+^/Ni in ChCl/EG stands at −0.154 V while that of Cu^+^/Cu is −0.350 V at 353 K since DESs provide diverse chemical environments compared with molecular solutions.^146^ Consequently, the galvanic replacement reaction between Cu and Ni^2+^ within ChCl/EG becomes thermodynamically favorable, enabling the fabrication of Ni thin films on the copper substrate.

#### 3.2.8 Intercalation and exfoliation

**Fig. 9:**
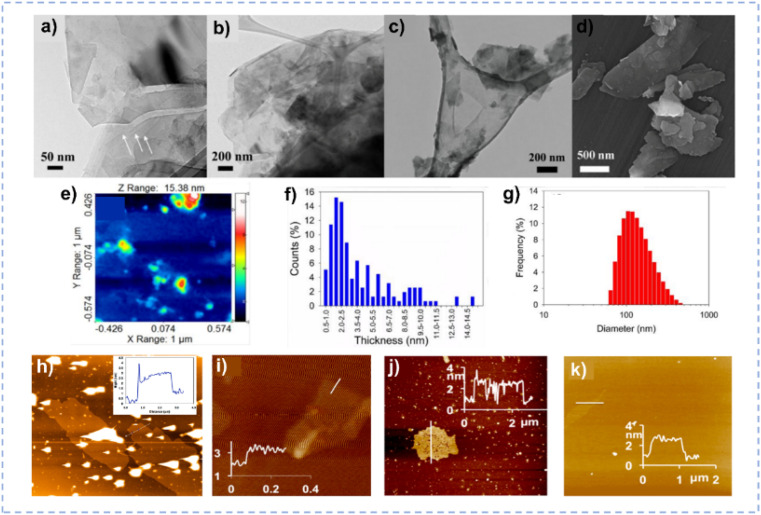
(a–c) TEM images of the exfoliated MoS_2_ nanosheets. (d) Field emission scanning electron microscopy image of the exfoliated MoS_2_. (e and f) AFM image and the corresponding thickness distribution of MoS_2_ nanosheets. (g) Particle size distribution of MoS_2_ nanosheets obtained by dynamic light scattering. Reprinted with permission from ref. 154 Copyright 2018, American Chemical Society. (h–k) AFM image and height profile for graphene (h), BN (i), MoS_2_ (j), and WS_2_ (k) after exfoliation. Reprinted with permission from ref. 35 Copyright 2016, American Chemical Society.

2D materials have abundant active sites and specific surface area, which is beneficial for catalysis.^148–150^ However, due to interlayer van der Waals interactions, the spontaneous stacking of 2D materials is inevitable, resulting in the reduced exposure of active sites.^151^ Owing to the distinctive hydrogen bond network and substantial ionic radius, DESs could be considered as effective intercalating and exfoliating agents, penetrating into the layers of materials to achieve exfoliation and obtain single-layer or few-layer 2D materials.^35,152,153^ Mohammadpour *et al.* demonstrated the remarkable efficiencies of sugar-based natural DESs for the liquid-phase exfoliation of bulk MoS_2_ (Fig. 9a–g).^154^ Through the size effect of sucrose molecules and the extended hydrogen bond interactions in the DES system, bulk MoS_2_ was exfoliated into 2D nanosheets with a yield of 44%.^151^ MoS_2_ nanosheets exhibit a mixed phase (2H–1T) with a 2H/1T ratio of 1.4. The exfoliated MoS_2_ shows an overpotential of 0.339 V *vs.* RHE at a current density of 10 mA cm^−2^ with long-term durability in acidic environments for the HER. Abdelkader *et al.* realized the exfoliation of 2D materials including graphene, BN, MoS_2_, and WS_2_ by the co-intercalation of Li^+^ and Et_4_N^+^ assisted by the ChCl/urea DES (Fig. 9h–k).^35^

### 3.3 DESs as electrolytes

DESs as electrolytes play pivotal roles in modulating the electron-transfer kinetics, facilitating mass-transfer kinetics, tuning the EDL interface structure, broadening ESW, changing the ion solvation environment, and suppressing side reactions in electrocatalytic reactions.^40,155–157^

The electrolyte plays a pivotal role in influencing the interfacial electron transfer process, thereby impacting the electrochemical kinetics.^158^ Zhen *et al.* investigated the electron-transfer kinetics in ChCl/EG s.^159^ The electron transfer rate constants observed in ChCl/EG are 100 times higher than those observed in the IL system. DESs show high dissolving ability for the reactants. For example, the reported solubility of CO_2_ in the prevalent ChCl-based DESs typically ranges from 0.28 to 0.60 mol_CO_2__ L^−1^ at 298 K and 1 atm. In contrast, the CO_2_ solubility is 0.03 mol_CO_2__ L^−1^ in aqueous solutions and 0.16–0.27 mol_CO_2__ L^−1^ in organic solvents under the same conditions.^157^ The excellent capture ability for CO_2_ increases the CO_2_ availability at the electrode surface, thereby benefiting CO_2_RR performance. However, despite the unique advantages of DESs in terms of electron transfer as well as improving the concentration of CO_2_ near the electrode, the current density obtained in the CO_2_RR using pure DESs as electrolytes is currently quite low. For example, in ChCl/EG, utilizing Au as the working electrode at −1.7 V *vs.* Ag/Ag^+^, the current density of the CO_2_RR is about 0.6 mA.^107^ Other DESs have similar problems, such as ChCl/urea, BmimCl/EG, ChCl/PEG, and BmimCl/PEG.^80^ Although close to 100% faradaic efficiency (FE) can be achieved for CO in DESs, the low current density due to the high viscosity restricts the application of DESs in electrocatalysis.^80^ Fortunately, current literature presents several potential strategies to address this challenge: (i) optimizing the electrochemical cell;^80^ (ii) modulating the electrode configuration and surface characteristics;^160^ (iii) developing DES-based electrolytes in non-viscous molecular solvents.^80,161^ As an example, the FE_CO_ increases from 15.8% to 59% just by increasing the amount of water in the viscous ChCl–urea DES.^80^ This increase in the catalytic activity is a consequence of the great reduction in the medium viscosity to facilitate mass transport. The introduction of water can disrupt the robust hydrogen-bond network inherent in the pristine DES, thereby enhancing the transfer of dissolved CO_2_, reaction intermediates and ions at the cathode surface. However, within a predominantly aqueous environment, the heightened availability of protons may concurrently elevate the FE for H_2_ production, attributable to the HER. To enhance the CO_2_RR while concurrently mitigating the competitive HER by diminishing proton availability at the cathode surface, non-aqueous solutions of DESs have been proposed, yielding exceptional outcomes. For instance, substituting ChCl–EG aqueous solutions with the dissolution of these DESs in acetonitrile has enabled an increase in current density during CO_2_ electrolysis from 0.4 mA cm^−2^ in pure ChCl–EG to 7.0 mA cm^−2^, alongside elevating the FE towards CO from 78% to 98.8%.^80^ This improvement stems from enhanced mass transport within these organic media and augmented CO_2_ availability at the electrode surface. Notably, the solubility of CO_2_ in organic solvents such as acetonitrile can reach levels up to eight times higher than in aqueous solutions, further contributing to the performance enhancements.^157^

**Fig. 10:**
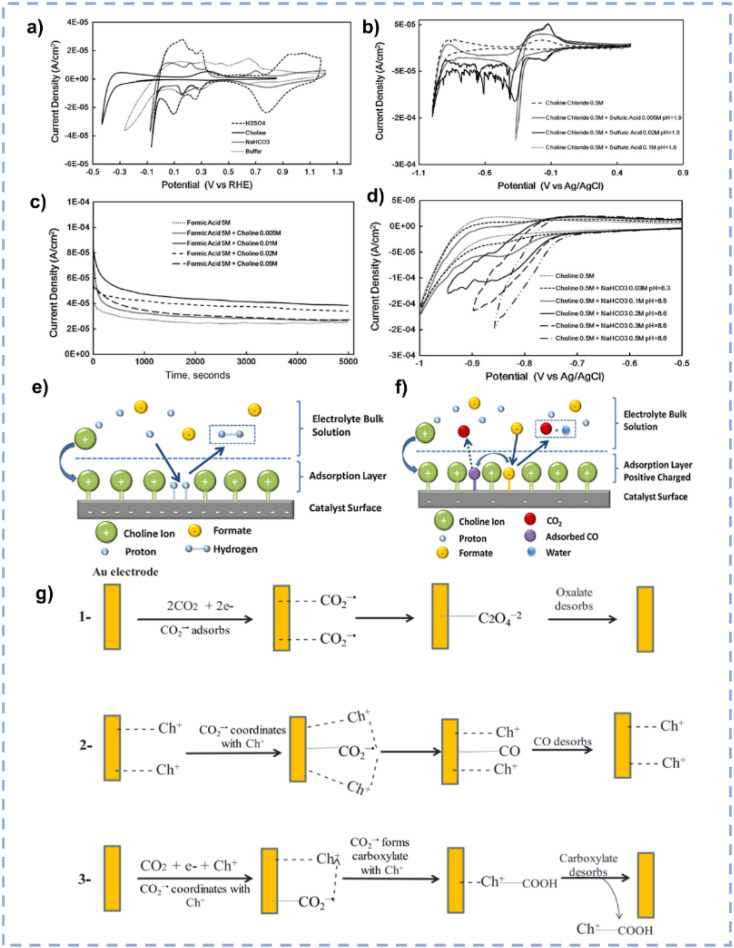
(a) CV curves over the Pt electrode in various electrolytes. (b) CV curves over the Pt electrode in various electrolytes with different pH. (c) Control potential electrolysis of formic acid electrooxidation over the Pt electrode in various electrolytes. (d) CV curves over the Pt electrode in various CO_2_-saturated electrolytes with different pH. (e and f) Schematic diagram for the influence of ChCl on the performance of the HER and electrooxidation of formic acid. Reprinted with permission from ref. 162 Copyright 2013, Elsevier. (g) Plausible CO_2_RR pathways over the Au electrode in ChCl/EG. Reprinted with permission from ref. 107 Copyright 2023, Elsevier.

In terms of tailoring the EDL structure, ChCl can play a crucial role in manipulating and stabilizing key intermediates.^162^ Zhu *et al.* investigated the effect of ChCl in the HER, formic acid electrooxidation, and CO_2_RR, respectively.^162^ For the HER in the ChCl-containing system, the cyclic voltammetry (CV) curve over the Pt electrode indicates the absence of characteristic hydrogen adsorption and desorption (Fig. 10a).^162^ The peak at 0.33 V *vs.* RHE is assigned to the interaction between choline ions and the catalyst surface.^162^ Moreover, a hydrogen reduction peak at −0.4 V *vs.* RHE suggests the inhibition of hydrogen production in the ChCl-containing system. Even with an acid environment, the HER is also suppressed in the ChCl-containing system (Fig. 10b). Without choline ion inhibitors, the surface is negatively charged when the potential exceeds the zero-charge potential, which facilitates proton adsorption on the electrode surface. In contrast, in the presence of choline cations, a thin layer of choline ions arrays on the catalyst surface (Fig. 10e). The choline cation layer results in a positive charge on the surface, reducing the proton concentration on or near the surface. The adsorption layer hinders the proton adsorption, lowering the reaction rate until the potential becomes negative enough for protons to eventually reach the catalyst surface. During the electro-oxidation of formic acid in the ChCl-containing system, a small quantity of ChCl can promote the oxidation of formic acid but cannot poison the catalyst (Fig. 10c). Formate can be stabilized through complexation with the positive charges in ChCl or hydrogen bonding with hydroxy protons in ChCl (Fig. 10f).^162^ In addition, compared with normal electrolytes, the ChCl-containing solution obtains a lower overpotential for the CO_2_RR (Fig. 10d). These results indicate that ChCl can act as a co-catalyst.^162^ Imteyaz *et al.* investigated the mechanism of the CO_2_RR over the Au electrode in ChCl/EG and proposed three plausible pathways (Fig. 10g):^107^ (1) the CO_2_^−^˙ undergoes dimerization on the Au electrode surface, generating oxalate salts; (2) the CO_2_^−^˙ interacts with choline cations adsorbed on the electrode surface and is then transformed into CO; (3) in the presence of DESs, the choline cation can stabilize CO_2_^−^˙, preventing its dimerization. High FE_CO_ is achieved, confirming that pathway (2) is more reasonable.

DESs can effectively reconfigure the hydrogen bonding network at the electrode interface to regulate the coordination environment of interfacial ions, inhibit side reactions, and promote ionic conduction. Wang *et al.* designed an acetamide–caprolactam DES-based electrolyte for stable Zn–metal batteries.^40^ This electrolyte reconstructs the hydrogen bond network in the electrolyte through acetamide (HBD) and caprolactam (HBA), effectively broadening the ESW and suppressing the reactivity of water that reduces the HER. Moreover, the coordination between Zn^2+^ and acetamide–caprolactam in DES-based electrolytes produces a unique solvation structure. Substantial enhancements in electrochemical stability and coulombic efficiency are realized by using DESs. The supramolecular hydrogen bonding network within DESs can facilitate proton conductivity in the solvent.^163^ Guo *et al.* designed a novel electrolyte, in which polyoxometalate nanoclusters are used as supramolecular cross-linkers to solidify levulinic acid/hydroxypropyl-β-cyclodextrin DES.^163^ A high proton conductivity (more than 1 × 10^−4^ S cm^−1^) at room temperature is achieved due to the abundant protons from polyoxometalate and the supramolecular hydrogen bonding network in the DES.

Despite the potential of DESs as electrolytes, to our knowledge, the current research based on utilizing DESs as electrolytes for electrocatalytic systems is still quite limited, and more studies are about the synthesis and preparation of electrocatalysts. This may be due to the high viscosity of DESs themselves leading to low current density during electrolysis. In addition, when some other small molecules are added to DESs to reduce the viscosity, the composition has already changed, and it is still questionable whether the mixtures under this composition are still DESs. When choosing the composition of DESs, it may be difficult for macromolecules or molecules with functional groups to form DESs. For alcohols, phenols, and carboxylic acid molecules as the composition of DESs, the potentials may lead to redox reactions of the components under different electrocatalytic reaction systems. Therefore, the current studies are very limited.

## 4. Applications of DESs in electrocatalysis

Electrocatalysis is an efficient way for the utilization of renewable energy to produce high-value chemicals, and significant progress has been achieved in electrolysis research over the past decades.^164–168^ The catalytic performance relies highly upon the electrocatalytic system, including the electrocatalyst and electrolyte, which determine the interface microenvironment and double-layer properties. Therefore, the construction of high-performance electrocatalytic systems by the synthesis of efficient catalysts and designing synergistic electrolytes is highly desirable. Thanks to the multiple functions of DESs (solvents, templates, structure-directing agents, precursors, and supramolecular networks), they are emerging as versatile media for building highly efficient catalytic systems. In this section, we summarized the recent developments in utilizing DESs for various electrocatalytic reactions.

### 4.1 HER

The HER represents the reductive half-reaction of water electrolysis, wherein protons obtain electrons to produce hydrogen.^169^ Although Pt is considered as the benchmark electrode for the HER, the high cost limits its widespread applications.^170^ Therefore, it is imperative to explore low-cost and high-performance electrocatalysts.

**Fig. 11:**
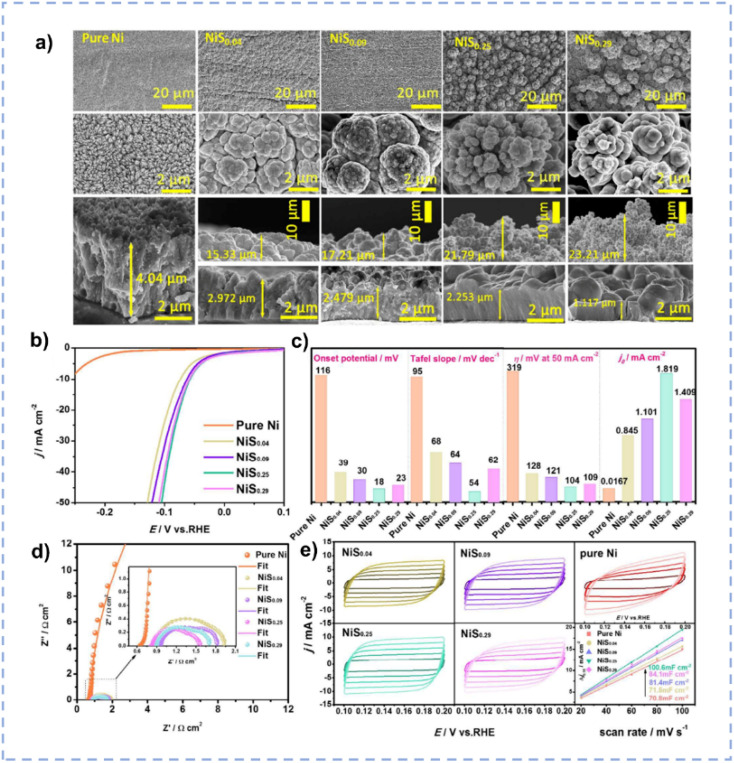
(a) SEM images of NiS_*x*_ with different amounts of S doping. (b) Polarization curves of pure Ni/CW and NiS_*x*_/CW with different amounts of S doping. (c) Comparison of the HER catalytic performance for the NiS_*x*_/CW with different amounts of S doping. (d) Nyquist plots of various NiS_*x*_/CW samples recorded at an overpotential of −110 mV. (e) CV curves over various NiS_*x*_/CW samples with different scan rates in a non-faradaic region and the corresponding capacitive currents as a function of scan rates. Reproduced from ref. 171 with permission from the Royal Society of Chemistry.

Nickel is a promising alternative to Pt for the HER, however, its robust hydrogen adsorption capacity results in a relatively slow desorption kinetics of H_2_.^30^ The introduction of S or P onto nickel can finely tune its electronic structure, optimizing both intrinsic activity and the adsorption free energy of H_2_. Zhang *et al.* prepared S-doped nickel microsphere films through an electrodeposition method directly onto copper wire in ChCl/EG.^171^ The incorporation of S induces a substantial amount of oxygen vacancies on the surface. The as-prepared materials with different Ni/S ratios exhibit different morphologies (Fig. 11a), and the NiS_0.25_ nanosphere exhibits the largest surface area. The synergistic effect between microsphere morphology and oxygen vacancies renders the highest electrocatalytic activity for the HER over NiS_0.25_ nanospheres (Fig. 11b–e). NiS_0.25_ shows a minimal overpotential of 54 mV to achieve a current density of 10 mA cm^−2^ and can be used for more than 60 hours in 1 M KOH.

The replacement reaction between Cu and Ni^2+^ can hardly occur in the aqueous electrolyte but can be realized in DESs as the redox potential of metals could be changed in DESs.^146^ Yang *et al.* fabricated Ni_3_S_2_@Cu *via* the galvanic replacement reaction, incorporating thiourea and Cu foil into the ChCl/EG–NiCl_2_ system.^79^ During the replacement reaction between Cu and Ni^2+^, S is incorporated into Ni films, resulting in the formation of S-doped Ni microsphere films on nanoporous Cu substrates. The Ni_3_S_2_@Cu exhibits significant electrocatalytic activity for the HER, confirmed by small Tafel slopes of 63.5 and 67.5 mV dec^−1^ in acidic and alkaline environments, respectively. Moreover, at a current density of 10 mA cm^−2^, the overpotentials in 0.5 M H_2_SO_4_ and 1.0 M KOH are 91.6 mV and 60.8 mV, respectively. Ni_3_S_2_@Cu exhibits higher catalytic activity than Ni nanocrystallites@Cu. Furthermore, multi-metallic catalysts applied to the HER have received increasing attention. By adding different metal salts to DESs, multi-metallic HER electrocatalysts can be prepared, such as Ni–Mo,^172,173^ Ni–Cu,^174^ Ni–Co–Sn,^175^ and Ni–Fe.^176^ These catalysts prepared using DESs presented lower Tafel slopes as well as low overpotentials.

### 4.2 OER

OER is the oxidation half-reaction of water spitting and usually exhibits slow kinetics due to the four-electron transfer process involved.^177^ Because the OER is the rate-limiting step in water splitting, the development of cost-effective and high-performance OER catalysts is necessary to improve the overall efficiency.^178^

**Fig. 12:**
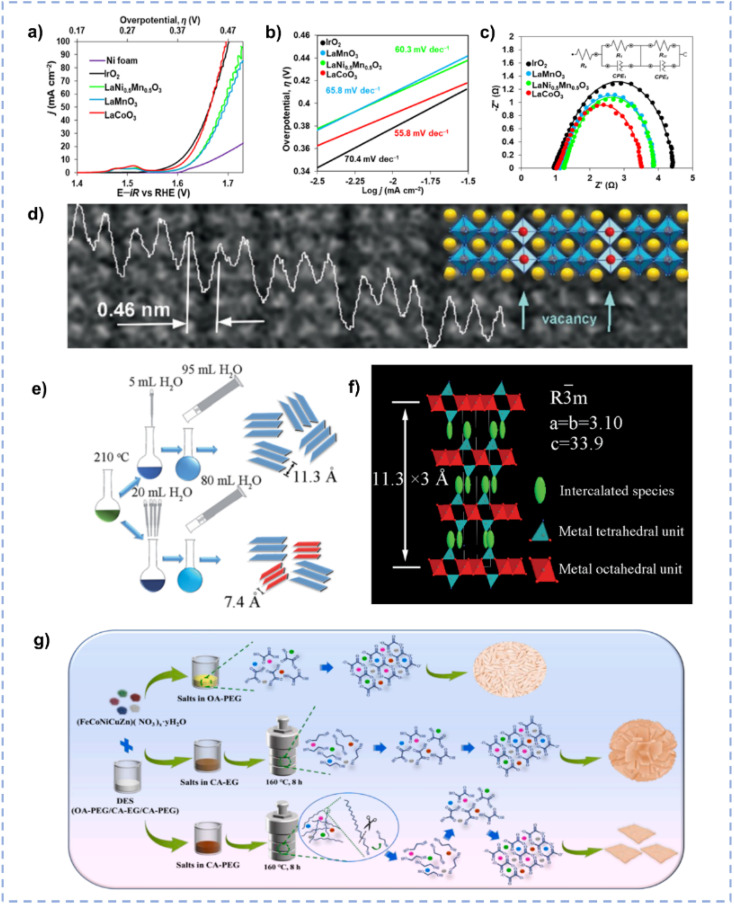
(a–c) Anodic polarization curves (a), Tafel (b), and Nyquist (c) plots over Ni foam-supported perovskites and reference IrO_2_. (d) Magnified HRTEM image with an overlaid structural model and intensity line scan profile. Reprinted with permission from ref. 181 Copyright 2022, American Chemical Society. (e) Schematic diagram for the synthesis of CoFe-LDH with different layer spacings. (f) The structure of interlayer intercalation of CoFe-LDH. Reproduced from ref. 184 with permission from the Royal Society of Chemistry. (g) Schematic illustration of the effect of DESs with different compositions on the final material morphology. Reprinted with permission from ref. 185 Copyright 2022, Elsevier.

Oxide perovskites, owing to their stability and tunable structure, have drawn significant attention as electrocatalysts for energy conversion.^179,180^ Hong *et al.* reported the synthesis of La-based perovskites using an environmentally friendly DES consisting of ChCl and malonic acid.^181^ The synthesis route involves the dissolution of the metal precursor in the DES followed by high temperature calcination, during which the high temperature leads to partial decomposition of the DES, resulting in the production of NH_3_ and HCl to boost the production of perovskite crystals. The DES-involved method realized the convenient, fast, and scalable synthesis of phase-pure crystalline materials compared to traditional solid-state methods. Among the as-synthesized perovskites (LaCoO_3_, LaMn_0.5_Ni_0.5_O_3_, and LaMnO_3_), LaCoO_3_ emerges as the best electrocatalyst for the OER in an alkaline medium (Fig. 12a–c). Current densities of 10, 50, and 100 mA cm^−2^ at overpotentials of approximately 390, 430, and 470 mV, respectively, are achieved over LaCoO_3_, and a Tafel slope of 55.8 mV dec^−1^ is obtained. The superior activity of LaCoO_3_ is ascribed to its high oxygen vacancy concentration (Fig. 12d), which is associated with the reducing atmosphere generated by the thermal decomposition of DES components.

2D materials have been widely used in the OER due to their large specific surface area and abundance of active sites.^182,183^ Ge *et al.* employed a “water injection” method to synthesize CoFe layered double hydroxide (CoFe-LDH) with an expansive layer spacing in ChCl/urea (Fig. 12e).^184^ Through the swift injection of water, urea and ChCl undergo decomposition, facilitating the formation of CoFe-LDH. In this procedure, derivative species originating from DESs serve as intercalators to generate large interlayer distances (Fig. 12f), resulting in good OER performance. The structure and composition of DESs exert influence on the nucleation and growth of multi-metal oxalate.^185^ The high molecular weight PEG and the gradual release of OA promote the formation of a 2D structure, whereas low molecular weight EG and ample OA favour the generation of 3D nanoparticles (Fig. 12g).^185^ The well-designed 2D (FeCoNiCuZn)(C_2_O_4_)·2H_2_O nanosheets exhibit an ultralow overpotential of 334 mV at 100 mA cm^−2^ for the OER with a prolonged durability of over 30 h. Hu *et al.* developed a one-step ionothermal-accompanied thermolysis method using DESs as precursors for the synthesis of ultrathin NiFe layered double-hydroxide hybridized nanosheets with N-doped carbon quantum dots (NCD@NiFe-LDH).^186^ The NCD@NiFe-LDH hybrid exhibits a hierarchical flower-like morphology, composed of 2D ultrathin nanosheets (∼1.4 nm thickness) with elevated surface area and excellent conductivity. This electrocatalyst for the OER demonstrates outstanding performance, requiring only 363 mV to obtain a high current density of 500 mA cm^−2^.

### 4.3 ORR

The ORR is a key reaction for fuel cells, and Pt-based electrocatalysts are commonly used for the ORR.^187–189^ To date, substantial endeavours have been dedicated to exploring cost-effective and superior electrocatalysts as alternatives to Pt-based catalysts. DESs can facilitate doping or act as a source of heteroatoms to prepare non-Pt-based high-performance catalysts. For instance, Luo *et al.* reported the facile preparation of nitrogen-doped graphitic carbon (NGC) *via* the pyrolysis of DESs.^190^ The NGCs have high surface areas, rich nitrogen content, and favourable graphitization degree and show excellent ORR performance compared with the commercial Pt/C catalyst. Pariiska *et al.* prepared a Co–N–C electrocatalyst by the pyrolysis of DESs containing 1-butyl-3-methylimidazolium chloride ([Bmim]Cl) or tetrafluoroborate ([Bmim]BF_4_) and hydrated Co(NO_3_)_2_ or CoCl_2_.^191^ DESs used as precursors can provide large amounts of Co and N atoms and promote the homogeneous dispersion of these elements. The Co–N–C shows comparable ORR performance with the Pt/C catalyst in alkaline electrolytes, and exhibit onset potentials of 0.96–0.99 V *vs.* RHE, half-wave potentials of 0.85–0.89 V and Tafel slopes of 37–50 mV dec^−1^.

### 4.4 CO_2_RR

**Fig. 13:**
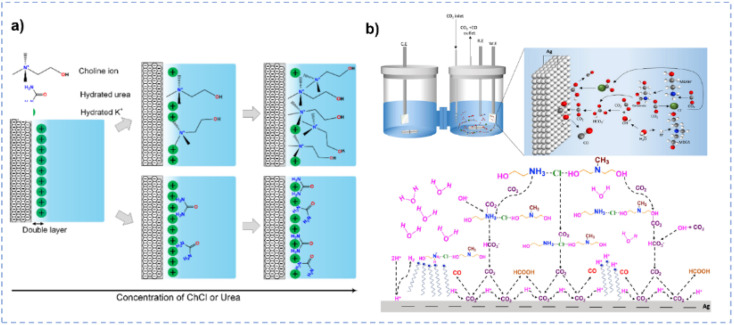
(a) The model of the double layer at the electrode interface. Reprinted with permission from ref. 194 Copyright 2020, John Wiley and Sons. (b) Mechanism for the CO production over the Ag electrode in MDEA-based DESs. Reprinted with permission from ref. 160 Copyright 2021, Elsevier.

CO_2_RR to high-value chemicals is a promising strategy for simultaneously realizing CO_2_ utilization and renewable energy conversion.^192^ Bohlen *et al.* prepared In coated Cu by electrodeposition in ChCl/EG.^193^ The as-prepared catalyst shows a FE_formate_ of ∼72.5%. Moreover, gas diffusion electrodes coated with In exhibit a formate concentration of ∼76 mM and a formation rate of 0.183 mmol cm^−2^ h^−1^. Garg *et al.* used ChCl/urea aqueous solution as an electrolyte for the CO_2_RR over a polycrystalline Ag foil electrode.^194^ FE_CO_ could reach 96% at −0.884 V *vs.* RHE in the 50 wt% ChCl/urea aqueous solution, surpassing the benchmark KHCO_3_ catholyte by over 1.5 times. The enhanced CO selectivity results from various ChCl/urea-mediated interactions with the Ag surface (Fig. 13a). First, the dissolution of the native Ag oxide layer in ChCl/urea, coupled with the re-electrodeposition of Ag nanoparticles under cathodic potential, leads to the reconstruction of the Ag surface, resulting in a high density of low-coordinated Ag atoms. These atoms exhibit strengthened bonds with intermediates and increase local pH. Second, choline ions adsorbed onto the surface hinder the proton migration that reduces undesired HER at the catalyst/electrolyte interface, and the –NH_2_ groups in urea facilitate CO_2_ reduction by providing an additional bond to the crucial *COOH intermediate that favors CO production. Ahmad *et al.* systematically conducted CO_2_RR in various aqueous solutions containing different amine-based DESs.^160^ The CO_2_RR process on the Ag electrode surface assisted with DESs is schematically illustrated in Fig. 13b, and the presence of DESs could affect the local microenvironment during the CO_2_RR. Specifically, methyldiethanolamine (MDEA)-based DESs in water not only produce protic species but also form HCO_3_^−^ by reacting CO_2_ with isolated OH^−^ ions or the –OH group in HBDs.^160^ Moreover, the –NH_2_ group in DESs is in the vicinity of the electrode surface, which affects the energy barriers for the generation and stabilization of the *COOH intermediate.^160^ Also, the equilibrium between dissolved CO_2_ and bicarbonate leads to an increase in the CO_2_ reduction rate. Particularly, [monoethanolamine hydrochloride] [methyldiethanolamine] ([MEAHCl][MDEA]) shows a high FE_CO_ of 71% at −1.1 V *vs.* RHE over the Ag electrode.

### 4.5 NRR

NH_3_ plays an imperative role in modern society by serving as a precursor to fertilizers and many other nitrogen-containing chemicals.^195^ Electrochemical synthesis of ammonia allows the thermodynamically non-spontaneous synthesis reaction of ammonia to proceed under mild conditions, overcoming or mitigating thermodynamic equilibrium restriction.^196,197^ N_2_ fixation is a kinetically sluggish and complex multistep reaction due to the high activation barrier for the cleavage of nitrogen–nitrogen triple bonds.^198,199^ In order to overcome the activation energy barrier of N_2_, Zhao *et al.* synthesized nanostructured Fe_3_S_4_ from the PEG 200/thiourea DES *via* a one-step solvothermal method.^200^ DESs can be used not only as directing agents but also as S sources to realize the formation of Fe_3_S_4_ nanosheets. The as-obtained Fe_3_S_4_ catalyst is capable of electrochemically reducing N_2_ to NH_3_ under ambient conditions and exhibits a high NH_3_ yield (75.4 μg h^−1^ mg_cat._^−1^) with a FE_NH3_ of 6.5% at −0.4 V *vs.* RHE. In addition, Chen *et al.* prepared a hybrid catalyst containing crystalline Fe_2_O_3_ and amorphous FeS using ChCl/thiourea as the solvent and S source.^201^ The DES exerts a structure-controlling influence on the development of nanostructures composed of small nanoparticles. Due to the large amount of exposed electrochemical active sites along with interfacial synergy between crystalline Fe_2_O_3_ and amorphous FeS, the as-prepared catalyst achieves a high NH_3_ yield of 34.31 mg h^−1^ mg_cat._^−1^ and a FE_NH3_ of 18.06% at −0.25 V *vs.* RHE during the NRR, outperforming most of the previously reported Fe-based catalysts. Mao *et al.* synthesized Pt–RE (RE = La, Y, Sc) alloy concave nanocubes (PtRENCs) with high-index facets (HIFs) by an electrochemical method in ChCl/urea.^202^ ChCl/urea not only exhibits excellent dissolving capacity for metal salt precursors but also promotes the generation of HIFs during electrodeposition through a unique solvent environment. The resultant Pt–La exhibits a unique electronic structure, leading to excellent NRR performance, with a NH_3_ yield rate of 71.4 μg h^−1^ mg_cat._^−1^ and FE_NH3_ of 35.6% at −0.2 V *vs.* RHE in 1 mM HCl.

Recently, Ying *et al.* synthesized Mn-doped Bi_2_O_3_ nanosheets in ChCl/OA.^143^ The ChCl/OA DES has excellent dissolving capacity for metal salts because OA can coordinate with metal ions.^115,142^ Mn-doped Bi_2_O_3_ nanosheets are prepared by microwave heating in ChCl/OA followed by calcination. Owing to the strong interaction between Bi 6p orbitals and N 2p orbitals, the competitive HER is evidently suppressed.^203,204^ Moreover, the coexistence of occupied and unoccupied 3d orbitals in the Mn element is conducive to adsorbing and activating N_2_ molecules.^205,206^ The as-prepared 5% Mn–Bi_2_O_3_ nanosheets achieve a high NH_3_ yield rate of 23.54 μg h^−1^ mg_cat._^−1^ and FE_NH3_ of 21.63% at −0.1 V *vs.* RHE.

### 4.6 Other reactions

#### 4.6.1 5-Hydroxymethylfurfural oxidation

Oxidation of 5-hydroxymethylfurfural (HMF) into 2,5-furandicarboxylic acid (FDCA) is a typical reaction for the transformation of biomass into valuable chemicals.^207,208^ Zhang *et al.* proposed a facile and controllable strategy for the preparation of carbon-based heterostructures by direct pyrolysis of (NiCl_2_·6H_2_O/CoCl_2_·6H_2_O)/PEG200/thiourea DES.^209^ The designed DES is considered as the metal source, N-source, S-source, and C-source. The simultaneous growth as well as the integration of metal sulfide and the carbon material occur in a single step, facilitating the uniformity of the composite. Moreover, numerous N, O, and S vacancies inevitably emerge during the calcination process. Notably, the S vacancies serve as anchoring sites for the formation of transition metal sulfide heterostructures. The as-prepared Co_9_S_8_–Ni_3_S_2_@N,S,O-tri-doped carbon heterostructures exhibit an excellent HMF electrooxidation performance, and FE_FDCA_ could reach 98.6% at a full conversion of HMF.

#### 4.6.2 Formic acid oxidation

Formic acid oxidation reaction has attracted great interest since the 1970s and is now widely investigated in fuel cells.^210–212^ Plaza-Mayoral *et al.* fabricated a Pd–Au bimetallic electrocatalyst by co-electro-deposition in ChCl/urea DES.^213^ Owing to the capacity of DESs for dissolving precursors and dispersing particles, the electrochemically active surface area (ECSA) of the Pd–Au electrocatalyst is 5 and 12-fold higher than those of Pd(poly) and PdAu(poly), respectively. Based on the high ECSA, Pd–Au alloys exhibit good activity and stability for formic acid oxidation. Yang *et al.* designed Pd_2_Ni_1_ nanocluster-supported multi-walled carbon nanotube (MWCNT) electrocatalysts in ChCl/OA.^214^ ChCl/OA serves as not only a solvent but also a reducing agent for synthesizing Pd_2_Ni_1_/CNTs, which exhibit four times higher activity for formic acid oxidation than the Pd/C catalyst. The high catalytic performance of Pd_2_Ni_1_/CNTs is ascribed to the special nanocluster structure and appropriate Ni doping, which changes the electron configuration of Pd to reduce the d-band and produce a Pd–Ni bond as a new active site.^214^

#### 4.6.3 Alcohol oxidation

Direct alcohol fuel cells have been extensively investigated for portable devices, primarily due to their high energy density and low toxicity.^215^ However, significant challenges, such as sluggish electrode reaction kinetics, limited long-term stability, and high cost hinder their commercial applications.^216^ Therefore, advanced alcohol oxidation catalysts need to be designed. DESs are effective in controlling the dispersion of nanoparticles and enhancing the electron transfer between the nanoparticles and the support material.^217^ Zhong *et al.* prepared hybrid PtCu nanoclusters on MWCNTs in ChCl/EG without adding any surface-controlling agent.^217^ The addition of alloyed Cu and ChCl/EG not only promotes the formation of nanoscale-structure with coarse surface but also decreases the particle size of metals. Moreover, the mass activity of PtCu/MWCNT is 2.5 times higher than that of Pt/MWCNT. Synthesis of electrocatalysts with high-index facets can be easily realized in DESs.^218^ Wei *et al.* synthesized Pt–Ru alloy concave nanocubes featuring a high-index (510) facet by the electrochemical square-wave potential technique in ChCl/urea DES.^219^ In comparison to both monometallic Pt concave nanocubes and quasi-spherical Pt–Ru alloys, the concave cubic Pt–Ru alloy with a high index facet shows outstanding electro-oxidation capability towards ethanol which is attributed to the abundant low-coordinated Pt sites (HIFs structure) and the distinctive electronic structure.

## 5. Summary and outlook

**Fig. 14:**
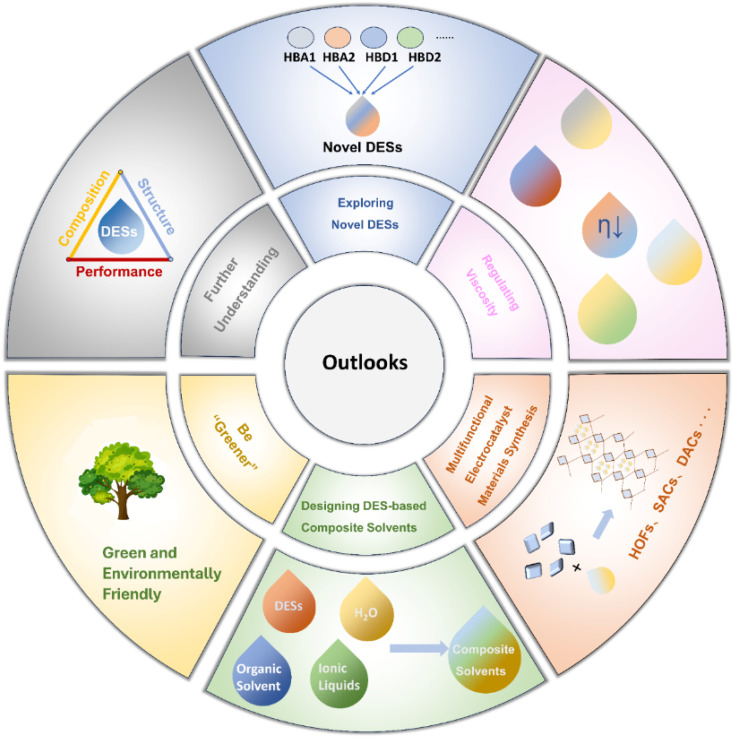
Prospects of DESs as solvents.

This review offers a thorough overview of the diverse applications of DESs in electrocatalysis. It encompasses an examination of DES features, highlighting their unique advantages as solvents for electrocatalyst synthesis and as electrolytes. Additionally, a systematic analysis is presented, detailing the typical applications of DESs in electrocatalysis. Finally, we endeavour to address challenges and provide insights into the future prospects of DESs in electrocatalysis, as illustrated in Fig. 14.

### 5.1 Be “Greener”

DESs have been regarded as green solvents to some extent and have found applications in various fields. However, some evidence suggests that DESs are not always environmentally friendly as initially thought.^220^ First, under harsh conditions, the hydrogen bonds between HBAs and HBDs in DESs can be disrupted. Additionally, chemical and electrochemical factors contribute to the instability of DESs. During the decomposition of DESs, evaporation may occur, resulting in the generation of toxic gases, environmental pollution, and the loss of atom economy. Furthermore, some DESs have been found to exhibit a certain degree of toxicity to biological tissues, including animals and plants.^221–224^ Given these challenges, it becomes necessary to explore strategies to enhance the green characteristics of DESs. Adjusting components, optimizing ratios, and incorporating stabilizing agents into DESs could help improve stability, reducing issues related to decomposition and evaporation, and ultimately lowering the environmental pollution risk.

### 5.2 Regulating viscosity

The high viscosity of DESs results in the restriction of mass transfer and reducing the reactivity of electrocatalytic systems significantly. The addition of water to DESs is the most commonly used method to reduce the overall viscosity. Here, we propose several plausible methods to reduce the viscosity of DES systems. (i) Precisely designing DES components from the molecular level. (ii) Introducing additives such as surfactants into DES systems.^225–227^ (iii) Adjusting the temperature of DESs during catalysis.

### 5.3 Exploring novel DESs

Current research in electrocatalyst preparation and electrolyte design mainly concentrates on previously developed DESs. As applications of DESs become increasingly extensive, the exploration of new DESs is very desirable. New combinations of intricate three-component or even four-component DES systems that involve various HBDs and HBAs should be prepared and systematically studied. The design of such multi-component DESs opens up more possibilities for synthesizing multifunctional electrocatalysts and employing them as electrolytes for various reactions. Solvent properties and reaction reactivity could be tuned by adjusting parameters such as chain length and functional groups of DES components. Furthermore, the study of the phase diagram is critical for the design and development of new DESs. However, current descriptions of DES phase diagrams are somewhat limited, with some studies lacking comprehensive information such as three-phase lines or focusing solely on eutectic points. It is anticipated that future research will provide more detailed insights into DES phase diagrams.

### 5.4 Designing DES-based composite solvents

DESs possess unique advantages but also exhibit certain drawbacks, such as higher viscosity than water and organic solvents and lower ion conductivity compared with ionic liquids. Since both solutions and solid materials belong to the condensed matter and share some similarities, especially from a functional design perspective, inspiration can be drawn from composite materials. In future research, DESs can be compounded with various solvents to create composite solvents, thereby leveraging the strengths of each component and mitigating their respective weaknesses. The multitude of possible combinations for composite solvents necessitates theoretical simulations to guide and reduce trial-and-error costs. Through the use of theoretical calculations and simulation methods, a deep understanding of the interactions between DESs and other solvents could be achieved.

### 5.5 Multifunctional electrocatalyst synthesis

DESs enable reactions under harsh conditions to proceed under mild conditions during material synthesis. Therefore, in the future, the exploration of DESs for synthesizing novel materials such as hydrogen-bonded organic frameworks (HOFs), single/dual-atom catalysts, high index facets, *etc.* as gentle reaction media is desirable. Considering that DESs could provide a stable reaction environment at low temperatures and pressures, along with their extensive supramolecular networks, they are poised to act as directing and templating agents for the synthesis of complicated materials. Besides, DESs are able to produce nitrogen atoms at certain temperatures for doping purposes, and they exhibit excellent dispersibility for metal ions. Consequently, DESs are potential solvents and precursors for preparing highly active and dispersive single/dual-atom catalysts.

### 5.6 Further understanding of composition, structure, and performance relationship

Until now, the understanding of the roles of DESs in regulating material synthesis and electrocatalysis remains limited. There is still some randomness in controlling the morphology and structure of catalysts. Further research into the involvement of DESs in the nucleation and growth of catalysts is necessary, accompanied by a thorough analysis of compositional change during the reaction. Recognizing and unveiling the structure–composition–performance relationship is crucial to achieve a rational and controllable design of DES-based catalysts. Future research would focus on tracking the surface reconstruction and microenvironment evolution during catalyst synthesis, which is beneficial for identifying real active species and mechanisms for specific reactions. The physicochemical properties such as viscosity, polarity, surface tension, hydrogen bonding, and aggregation or phase behaviour of DESs should be focused. These essential parameters are essential for influencing the reactivity and mass transport to determine the structure–performance relationship.

In summary, suitable DESs could provide distinct advantages over both aqueous solutions and IL-based solutions within electrocatalytic reaction frameworks. Their unique hydrogen bonding networks, phase behaviour, and broad ESW endow DESs with unparalleled functionality in material synthesis and electrolytes. This encompasses a wide range of capabilities, including morphology control, defect engineering, elemental doping, intercalation and exfoliation, modulation of metal redox potentials, and acceleration of synthesis kinetics. Moreover, as electrolytes, DESs effectively manage the EDL interface and ionic solvent environments, while also suppressing side reactions and stabilizing key intermediates. Despite their widespread application in various electrocatalytic reactions such as the HER, OER, ORR, NRR, CO_2_RR, *etc.*, the exploration of DESs remains relatively limited and necessitates further expansion. Future endeavours should prioritize the design of more robust DESs, optimization of viscosity for enhanced mass transfer, innovation of novel DES compositions, fabrication of composite solvents, engineering of nanomaterials utilizing DESs, and elucidation of the structure–performance relationship of DESs. These avenues represent critical directions for advancing the understanding and application of DESs in electrocatalysis.

## Author contributions

Hengan Wang: investigation, conceptualization, visualization, writing – original draft. Xinchen Kang: supervision, writing – review & editing. Buxing Han: supervision, writing – review & editing.

## Conflicts of interest

There are no conflicts to declare.
